# Assessing tele-manipulation systems using task performance for glovebox operations

**DOI:** 10.3389/frobt.2022.932538

**Published:** 2022-11-25

**Authors:** Erwin Jose Lopez Pulgarin, Ozan Tokatli, Guy Burroughes, Guido Herrmann

**Affiliations:** ^1^ Department of Electrical and Electronics Engineering, The University of Manchester, Manchester, United Kingdom; ^2^ Remote Applications in Challenging Environments, United Kingdom Atomic Energy Authority, Abingdon, Oxfordshire, United Kingdom

**Keywords:** task performance, bilateral teleoperation, robotic glovebox, robotics, experimental validation, nuclear robotics, radiation surveying

## Abstract

Tele-manipulation is indispensable for the nuclear industry since teleoperated robots cancel the radiation hazard problem for the operator. The majority of the teleoperated solutions used in the nuclear industry rely on bilateral teleoperation, utilizing a variation of the 4-channel architecture, where the motion and force signals of the local and remote robots are exchanged in the communication channel. However, the performance limitation of teleoperated robots for nuclear decommissioning tasks is not clearly answered in the literature. In this study, we assess the task performance in bilateral tele-manipulation for radiation surveying in gloveboxes and compare it to radiation surveying of a glovebox operator. To analyze the performance, an experimental setup suitable for human operation (manual operation) and tele-manipulation is designed. Our results showed that a current commercial off-the-shelf (COTS) teleoperated robotic manipulation solution is flexible, yet insufficient, as its task performance is significantly lower when compared to manual operation and potentially hazardous for the equipment inside the glovebox. Finally, we propose a set of potential solutions, derived from both our observations and expert interviews, that could improve the performance of teleoperation systems in glovebox environments in future work.

## 1 Introduction

Nuclear decommissioning is one of the biggest challenges faced by the nuclear industry and governments around the world. The United Kingdom has the largest nuclear decommissioning and remediation programme in Europe, and the current plan to decommission the legacy nuclear facilities will take a hundred years and cost billions of pounds (Nuclear Decommissioning Authority, 2021).

Nuclear gloveboxes are an integral part of the decommissioning tasks, where contaminated objects are handled by professional operators. In glovebox operations, the radiation hazard for the operator is lowered but not completely eliminated. On a few occasions, operators were exposed to radiation as a result of an accident with the glovebox (Rollow, 2000; Hagemeyer and McCormick, 2012; Cumbria, 2019). The risk of accidents forces operators to adopt strict operational measures. Moreover, gloveboxes are unergonomic by their designs, and as a result, working in a glovebox is a strenuous job for the operators.

There are various challenges in nuclear decommissioning, and gloveboxes are identified as a case study for implementing robotic technology for manipulation by 2025. While the goal is to implement a bilateral teleoperation system for performing some of the decommissioning tasks in a glovebox, the current vision is to take over 50% of the glovebox operations from human operators by 2030 (Nuclear Decommissioning Authority, 2021).

Teleoperated robots offer safer manipulation in hazardous environments by keeping the operators away from radiation sources and allow operators to continue working on their tasks without being limited by the levels of the exposed radiation. Moreover, teleoperated robots with assistive control techniques can potentially improve the performance on decommissioning tasks. However, despite the common use of robotic teleoperation in nuclear applications, the performance levels of bilateral tele-manipulation systems are not clear and often difficult to measure.

Understanding the task performance in tele-manipulation is crucial for designing better and more capable robotic systems for nuclear decommissioning. However, evaluating the performance could be challenging since comparing two different manipulation methods faithfully cannot always be achieved by objective metrics, especially when humans are involved in the manipulation process. Therefore, objective measures and subjective assessments should be used coherently to understand the task performance. To the best of our knowledge, no systematic task performance assessment of teleoperated robots used in nuclear sites has been carried out. This study aimed to open a new perspective on understanding the performance offers of teleoperated robots in nuclear operations.

In this study, manual object manipulation, i.e., using hands directly for manipulating objects, is assumed as the most intuitive and easy-to-use manipulation method for humans, and it is treated as the ground truth for our study.

Understanding the limitations of bilateral teleoperation-based robotic systems is of great interest due to their current popularity in the nuclear industry. This study aimed to evaluate the performance of a bilateral tele-manipulation system working in a nuclear glovebox for a realistic task to understand the limitations of such teleoperated robots. As these systems are based on the well-known 4-channel architecture, where the motion and force signals of the local and remote robots are exchanged in the communication channel (Lawrence, 1993), the results can be extended to systems with a similar architecture. Moreover, the performance of the tele-manipulation system is compared to the human manipulation inside the glovebox, which is referred to as manual manipulation. A manipulation system which includes human intervention is hard to assess objectively as the operators vary in every respect such as knowledge on the system and dexterity in manipulation. Therefore, this study proposes an experimental setup which is refined from a realistic task performed daily by professional operators in the nuclear industry. Objective metrics are used for measuring the performance. The main contribution of this study is to provide a clear understanding of the performance shortcomings of glovebox bilateral tele-manipulation systems for real-life manipulation tasks. Authors have hypothesized that due to factors such as lack of sensory information and use of unintuitive kinematic structure of the local (master) device, the performance of robotic tele-manipulation is worse than that of manual manipulation, and it places a higher cognitive load on the operator. To support our experimental performance evaluation approach, we interviewed our participants who are nuclear industry professionals, and their subjective views on the tele-manipulation system are presented.

The paper is organized as follows. Section 2 presents the related work on the problem of evaluating the performance in teleoperated robotics. Section 3 explains the experimental setup, the design of the experiment is presented, and performance metrics. Section 4 presents the results of the experiments, and Section 5 provides a discussion of the results. Finally, Section 6 concludes the paper.

## 2 Related work

### 2.1 Performance in tele-manipulation

Research on assessing the performance of teleoperated robotics can be organized into two groups: system or device performance and task performance. The system performance refers to the quantitative analysis of the robotic device used in the tele-manipulation (e.g., robotic arm). In contrast, task performance considers the user, the device, and the task execution simultaneously to obtain metrics relevant to a use case or application. Despite the importance of the manipulation interface, this work focused on the operator and its interaction with the robotic device while completing a task.

The literature on teleoperated robots has a wide spectrum of task performance analyses; however, comparing tele-manipulation to manual manipulation has drawn less attention. Richard et al. (1999) considered the performance in the pick-and-place task for teleoperated robots with different feedback modalities and manual task execution. In order to obtain reproducible results, the teleoperated system was implemented in a virtual environment, and the operator used a haptic interface for manipulation. It was shown that task completion time and accuracy were better in manual manipulation, whereas force feedback improved the accuracy in teleoperation.

In a different application area, the task performance of teleoperated robots and manual manipulation was given by Li et al. (2000). Experienced surgeons were asked to perform suturing using conventional open surgery, with laparoscopic tools, and finally, with teleoperated surgical robots. It was shown that suturing with teleoperated robots took longer to complete than conventional methods, with a higher leakage rate. However, it was found that teleoperated robots provided better performance than laparoscopic tools due to the lack of the fulcrum effect.

Motor skills play a key part in manipulation, and being able to assess the human motor skill capability is an important measure of the task performance. Geiger et al. (2010) developed a new test bed for assessing the fine motor skills with teleoperated robots. The aiming, finger dexterity, manual dexterity, and wrist-finger speed were evaluated. It was found that, compared to human hand assessment, teleoperated robots increase completion times. Moreover, all the evaluated factors were negatively influenced by the teleoperation system.

In the context of this study, teleoperation with dissimilar kinematics is an important concept to understand. In this teleoperation setting, the master and slave robots have different kinematic and, possibly, dynamic characteristics; therefore, the control modality for this type of teleoperation differs from the control modality of similar kinematic teleoperation. Pan et al. (2017) addressed some of these challenges, focusing on solutions that include a model of the remote robot and its environment to solve problems such as synchronisation (Xu et al., 2016) and controllability. Such methods include the use of digital fixtures to deal with dissimilar kinematics (Liu et al., 2019), which effectively simplifies the control task but for which the task performance is still challenging. Ben-Porat et al. (2000) investigated the task performance analysis with dissimilar kinematics for surgical application. The authors had investigated the placement of the master robot and the visuo-motor mapping of the remote environment. It was found that master robot placement has a direct effect on the task performance. Moreover, it was shown that simplifying assumptions on complex tasks could give misleading results on the actual task performance.

Another study that focused on the effect of force feedback on task performance was performed by Wagner and Howe (2007). The performance of a teleoperated robot for a surgical task was evaluated, and it was found that the force feedback reduces the forces applied in the remote environment. It was also found that force feedback reduces the completion times for trained surgeons; however, novice users did not benefit from the haptic feedback. Similar results about the trade-offs of using force feedback were obtained by Wang et al. (2018) when assessing the performance of different control algorithms for robotically steered needles, with force feedback improving the performance when used with Cartesian control but increasing user cognitive load and muscular fatigue.

Yip et al. (2011) evaluated the task performance for a simple peg-in-the-hole task with a time delay in the force signal. Different teleoperation modalities, such as unilateral, bilateral, and no force feedback with various time delay levels, were considered. It was shown that time delay increases task completion times regardless of the teleoperation modality. Force feedback reduced the force applied to the remote environment.

### 2.2 Robots in gloveboxes

Gloveboxes are protected environments used in many applications [i.e., chemical pharmacy (Perera et al., 2018) and nano-particle production (Masubuchi et al., 2018)] to create, protect, and isolate an area that contains sensitive or dangerous substances during a productive process. In applications such as civil nuclear, they are used by operators to protect them while manipulating hazardous objects during a maintenance task. Using robots for glovebox operations has long been an interest for the robotics community (Pedrotti et al., 1991; Grasz and Perez, 1997). Akiyama (1996) used a robotic manipulator as an early example of robotic decommissioning, where the robot was used to dismantle the JDPR reactor. An autonomous robotic system is proposed in Harden et al. (2009) to reduce the radiation hazard for the operator and to improve safety. Similarly, robotic systems are proposed for reducing operational costs (Peterson, 2000; Sands, 2006). Robots inside a glovebox are affected by hazardous radiation, reducing their shelf life and functionality, with mitigating techniques such as the one studied by O’Neil et al. (2012), which moves the robot away from areas of larger danger. However, there are individual differences among operators using teleoperated robotic glovebox systems, which include preferences for improved perceived comfort and for arm trajectories of robotic manipulators during operation (Sharp et al., 2017).

Redundant manipulators have been extensively used in gloveboxes for improving the manipulation capability inside the contained space. Turner et al. (2001) used a redundant manipulator to avoid collisions with the environment. Another use of redundant manipulators in gloveboxes includes improving the manipulation (Roa and Suárez, 2015) for robust handling of objects.

The glovebox operations are physically demanding for the operators who are exposed to radiation hazards. Therefore, not only the operator’s safety is in question, but performance limitations in glovebox operations also pose a challenge on their own. Despite extensive training aimed at improving the operator’s skills for executing glovebox operations, robotic systems have been shown to provide assistance to operators (O’Neil, 2010). Ghosh et al. (2020) investigated the use of high-level voice commands for humanoid robots which are designed to work in legacy gloveboxes. Finally, other robotic applications for gloveboxes include the use of humanoid robots (Long and Padir, 2018; Onol et al., 2018) and continuum robots (Lastinger et al., 2019) to help perform maintenance tasks in constrained environments such as a glovebox.

## 3 Experimental performance evaluation

### 3.1 Performance evaluation methodology

The main focus of this work is to identify the performance of different manipulation methods in the context of a realistic task performed in daily glovebox operations. The general methodology for evaluation followed these four points:

• Identify the realistic task. Preferably, the task should be an integral part of the daily operation routines.
• Identify the performance measures that indicate a successful and efficient task.
• Design an experiment around the task. The experiment should promote repeatability and highlight the essence of the realistic task.
• Perform controlled and randomized experiments with trained test subjects.

An experimental approach was used to understand and evaluate the performance of bilateral tele-manipulation for glovebox operations, considering task performance as its main component. For this, we first selected a representative task performed in glovebox operations, and then we created a parametrizable test based on the task itself to be applied under different manipulation methods (i.e., manual operation or tele-manipulation). Performance metrics were derived based on expected timings, accuracy, and possible effects that an unplanned interaction with the glovebox would provoke (e.g., unplanned collisions with the glovebox and cross-contamination).

The task for the experiments is radiation surveying, which is common in many glovebox operations and often performed by trained operators. In this work, we refer to tasks as a sequence of operation-relevant activities performed during a glovebox operation (e.g., opening and closing a sealed container are tasks performed during a maintenance operation). Basic actions such as grasping or moving an object are interpreted as building blocks of a task performed in a glovebox operation.

This general methodology can be adapted to other tasks. However, our results should be indicative of the expected performance while performing other tasks for the same tele-manipulation system, as most tasks performed inside a glovebox face the same limitations (i.e., use of tools to interact with substances while suffering from poor visibility and dexterity).

### 3.2 Radiation surveying in glovebox operations

In the context of glovebox operations, radiation surveying is a task performed to know the levels of radiation in any potentially contaminated object or area by using a handheld sensor (probe) connected to a ratemeter outside the glovebox. The sensor’s response to ionizing radiation, such as alpha and/or beta particles, is dependent on its distance from and relative orientation to the source. The main difficulties faced by trained operators while performing radiation surveying are around manipulating the handheld sensor at a constant distance to the source while slowly moving the probe to avoid collisions as it could damage or contaminate the probe. Moreover, the operators perform radiation scanning while wearing multiple layers of protective gloves, under reduced mobility and dexterity due to the limitations of the glovebox and under reduced visibility of the tinted glasses of the glovebox, which makes it difficult to estimate the clearance between the probe and the source.

Radiation surveying tasks can be classified into two groups: 1) an operator surveying a visible object (object radiation surveying) and 2) an operator discovering potential radiation sources or contaminants in the glovebox (workspace radiation surveying). Object radiation surveying is often the main focus of glovebox operations, where an object of unknown levels of radiation is taken out of its specialized container and surveyed to understand its level of degradation over time. Workspace radiation surveying is a task where the interior of the glovebox is surveyed to identify residual levels of radiation.

Nuclear gloveboxes are *active* work environments, as they are regularly used for decommissioning and maintenance tasks. As a result, particular areas and surfaces in the glovebox can become active with residual levels of contaminants. To contain the contamination and minimize the radiation hazard, workspace radiation surveying has to be securely and reliably conducted by the operator.

### 3.3 Radiation surveying testing protocol

#### 3.3.1 Test description

An experiment was designed based on workspace radiation surveying, as defined in Section 3.2. A repeated measure experimental design was used to quantify and measure the task performance of two manipulation methods (manual operation and tele-manipulation).

**FIGURE 1:**
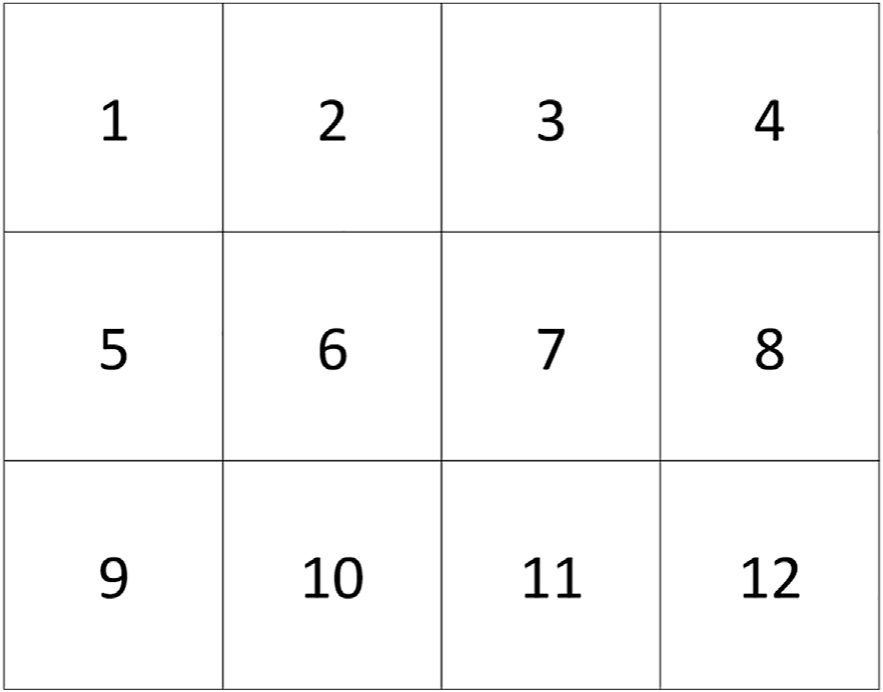
Physical layout for a radiation surveying test.

**FIGURE 2:**
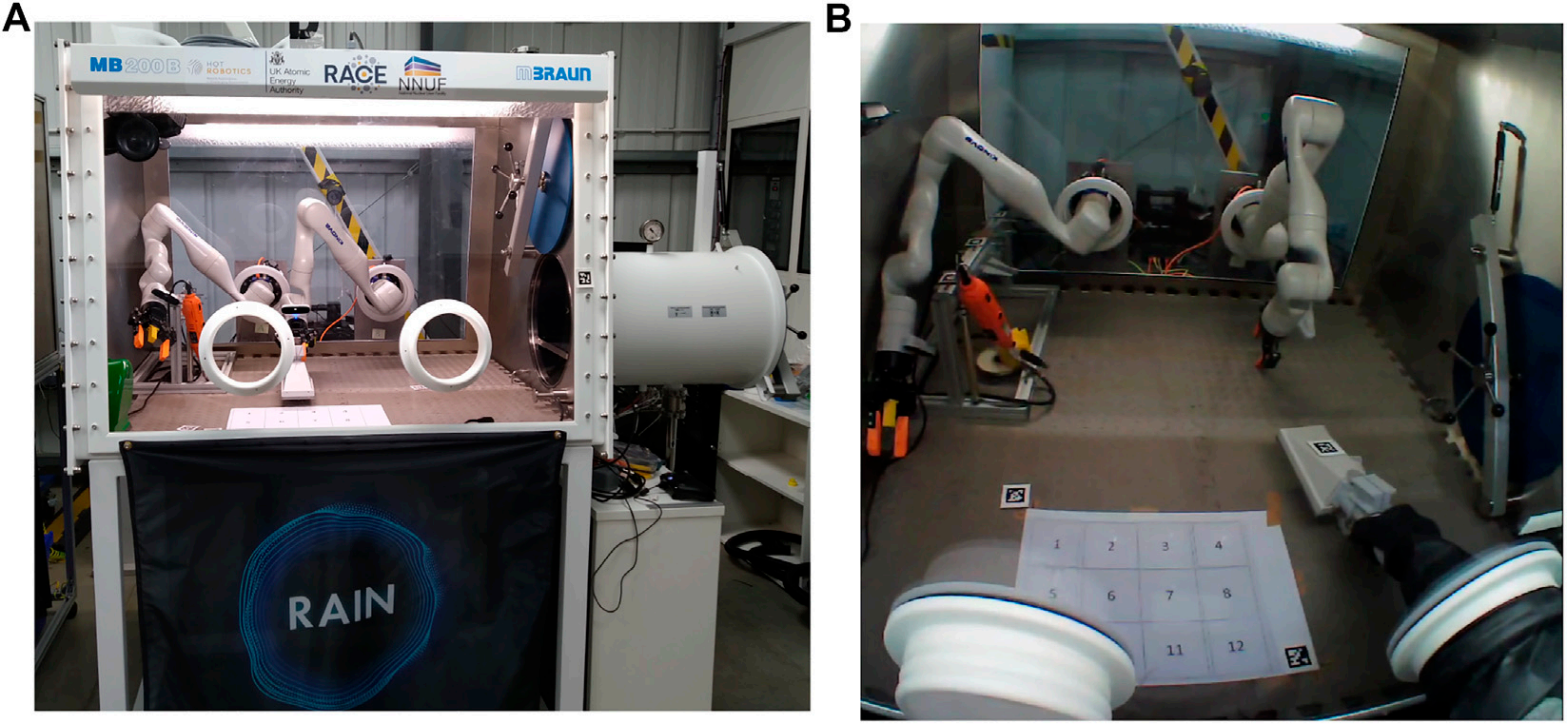
**(A)** Glovebox used for experiments. The remote environment is manipulated using the Kinova Gen 3 robotic arm in the right. The glove ports are used for accessing the glovebox interior. **(B)** Glovebox workspace during experimental trials. The grid is placed closer to the operator and robots are turned off during manual manipulation trials.

In the experiments, the test subject looked for an unknown simulated radiation source inside the glovebox workspace and reported it to the experimental officer. The glovebox workspace was defined as a 30 cm × 40 cm area and segmented in sectors of 10 cm × 10 cm, as shown in Figure 1. The number of contaminated sectors was unknown to the test subject, and it varied from one to four tiles per iteration. Each participant repeated the task four times, with each iteration being timed and their answers recorded. A glovebox with multi-robotic manipulator capabilities was used (Figure 2A), but only the right robotic manipulator was used. The glovebox workspace was located in front of the glovebox’s glove port for manual operation and inside the robot’s workspace, as seen in Figure 2B.

An STS Ionizing Radiation Simulator System was used to simulate a contaminant in the glovebox workspace being detected by a probe. The DP6-RE Simulated Probe (STS Ltd, 2022) and the Thermo RadEye SX radmeter were used with the liquid simulated source LS1, which produces a gas when in contact with the air that the probe identifies as ionising radiation. The radmeter alerts the user of changes using its display, a red LED and a sound alarm. The LS1 liquid was sprayed on tiles placed in any of the sectors of the glovebox workspace, changing its location in-between tests. The DP6-RE Simulated Probe was fitted with a gripping block that enables a robot to grasp it easily. This equipment is used to train operators in cross-contamination and decontamination exercises, as it simulates the size and behavior of a real probe (i.e., it needs to be moved close to and slowly over a surface).

The experiment began with a general explanation of the task to be performed, the sensors and measurements taken during each trial, and a short familiarisation stage with the simulated radiation sensor and the robotic manipulation method. Test subjects performed the task manually and then switched to using the tele-manipulation setup (see Section 3.3.3). At the end of the experiment, a NASA-TLX test (Hart and Staveland, 1988) was carried out, and a short interview was conducted to understand the view of the participant on the preferred manipulation method.

#### 3.3.2 Test subjects

Test subjects were recruited from a pool of individuals working or associated with the Remote Applications in Challenging Environments (RACE) of the United Kingdom Atomic Energy Authority (UKAEA). The recruitment process primarily focused on research engineers working in the area of teleoperated robots and remote handling operators who are experienced on remote maintenance tasks inside gloveboxes or in similar environments. Moreover, experience in robotics was required for the participants, and the participation in the experiment was not imposed on the test subjects as a requirement for their continuous employment. All health and safety requirements were met to operate the devices, and risk assessments were prepared for operating all the equipment used in the experiment. The national guidelines for close contact with test subjects were followed during the experiment. No ethical approval was required as no personal, sensitive, or confidential data were acquired, with the consent given by the participants. The data recorded during the trials were anonymised and kept away from third parties. Individual views were not shared with anyone outside of the authors of the work and were only grouped for further reporting.

#### 3.3.3 Manipulation methods

In the experiment, two different manipulation methods were used to assess the operator’s performance in the radiation survey task, as described in Section 3.3.1.

##### 3.3.3.1 Manual manipulation

A radiation probe for measuring the (simulated) radiation level on a surface was used by the subject, who was told to use their dominant hand while wearing a pair of protective gloves. Figure 2B captures an exemplary trial with a subject using the probe on the grid given in Figure 1.

##### 3.3.3.2 Tele-manipulation

In the experiment, a commercial off-the-shelf bilateral tele-manipulation system was used to perform glovebox tasks. On the remote side, the tele-manipulation system consists of a Kinova Gen3 robotic arm with 7 degrees of freedom (DoF), mounted with a Robotiq 2f-85 parallel jaw gripper with modified fingers. The remote robot was fixed to a pedestal outside the glovebox and fed into the glovebox through the existing glove port, as seen in Figure 2B. The Kinova Gen3 robot has been used to develop a robotic glovebox (Tokatli et al., 2021), as they are of low cost and of ideal size to fit through the entry port of most gloveboxes.

**FIGURE 3:**
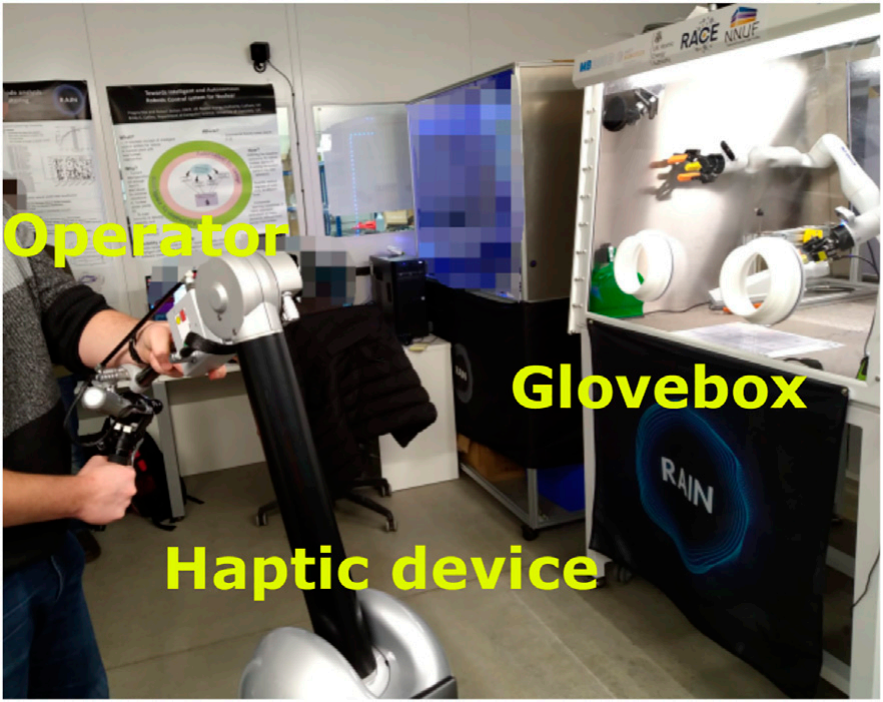
Test subject operating the Haption device to perform radiation surveying.

The local side of the tele-manipulation system is a Haption Virtuose™ 6D haptic interface (Garrec et al., 2004). The haptic interface allows intuitive control of the remote robot’s end-effector; hence, it requires a minimal amount of familiarization with the teleoperation system. Moreover, with the help of the buttons on the haptic interface, the operators can adjust the remote robot’s elbow configuration using the redundancy available in the remote robotic arm. This is achieved by utilizing the null space of the redundant robot’s kinematics, where the joint space motion is projected into the null space of the kinematic Jacobian of the redundant manipulator; therefore, the end-effector position is kept unchanged while the robot joints move (Siciliano et al., 2008). This kind of motion is useful during manipulation to avoid collisions. The test subjects held the haptic interface 0.5 m away from the glovebox itself, with a clear view over the workspace as seen in Figure 3.

The choice of this particular teleoperation setup is based on its availability. The Haption Virtuose haptic interface is a commercial solution that is being adopted in many teleoperation and remote handling applications and provides high force feedback in six DOF with out-of-the-box integration with many robots, including the Kinova Gen3 robotic arms. The control system and the inverse kinematics of the tele-manipulation system were used as supplied by the vendor.

#### 3.3.4 Sensors and performance metrics

In order to identify the task performance of each subject, three task performance metrics are defined.

• *Task accuracy:* identifying the cell number(s) contaminated with simulated radiation,
• *Completion time:* which is measured in seconds, and
• *Occurrence of a collision* between the sensor and the environment.

The performance metrics are designed considering the objective of a radiation surveying task. A successful radiation survey should identify the hot spots in the environment without previous knowledge, and during the surveying, the probe and the potentially contaminated surface should not collide.

In addition to the performance metrics, additional sensory information was gathered during each experimental trial to understand both the robot and human behavior during each trial and how these change per the manipulation method. Three video feeds were recorded with a timestamp overlay to aid analysis, two from the inside of the glovebox (side and top view) and one from the test subjects while performing the task. Eye tracking glasses were used (Tonsen et al., 2020) with fiducial markers on the scene to record gaze patterns and compare them between manipulation methods.

### 3.4 Hypothesis

It would be expected that an appropriate teleoperation method allows for a task to be performed similarly or better than when performed manually. However, we expect that compared to manual operation, tele-manipulation would make a task take longer due to it being harder to control.

## 4 Experimental results

**FIGURE 4:**
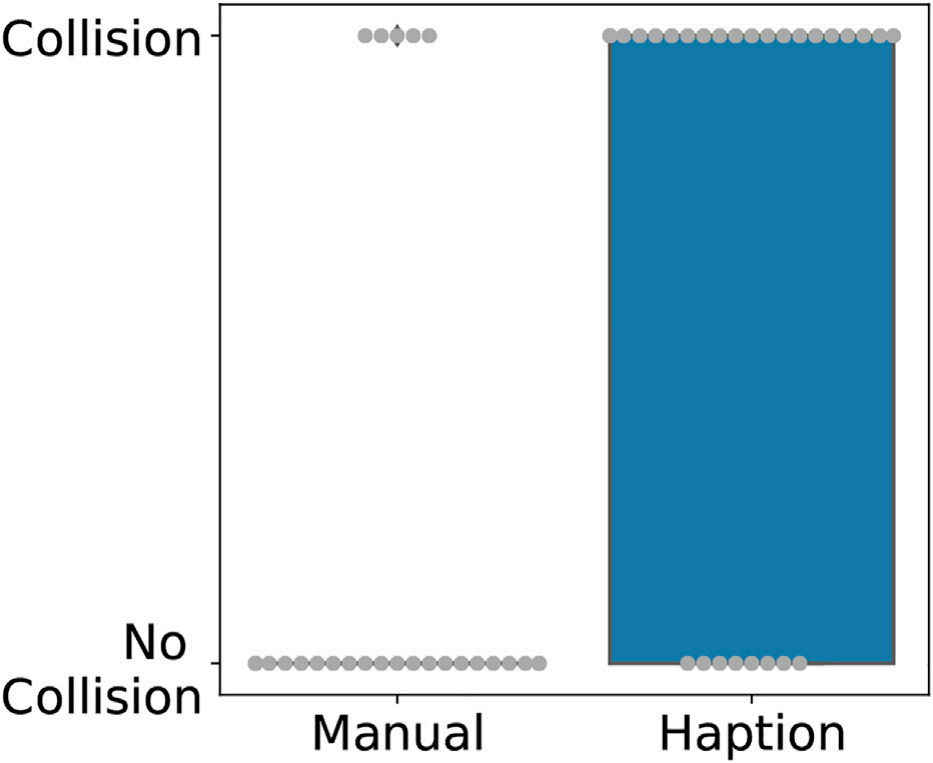
Boxplot with overlaid beeswarm plot comparing the occurrence of collisions between the probe and the glovebox floor per trial.

**FIGURE 5:**
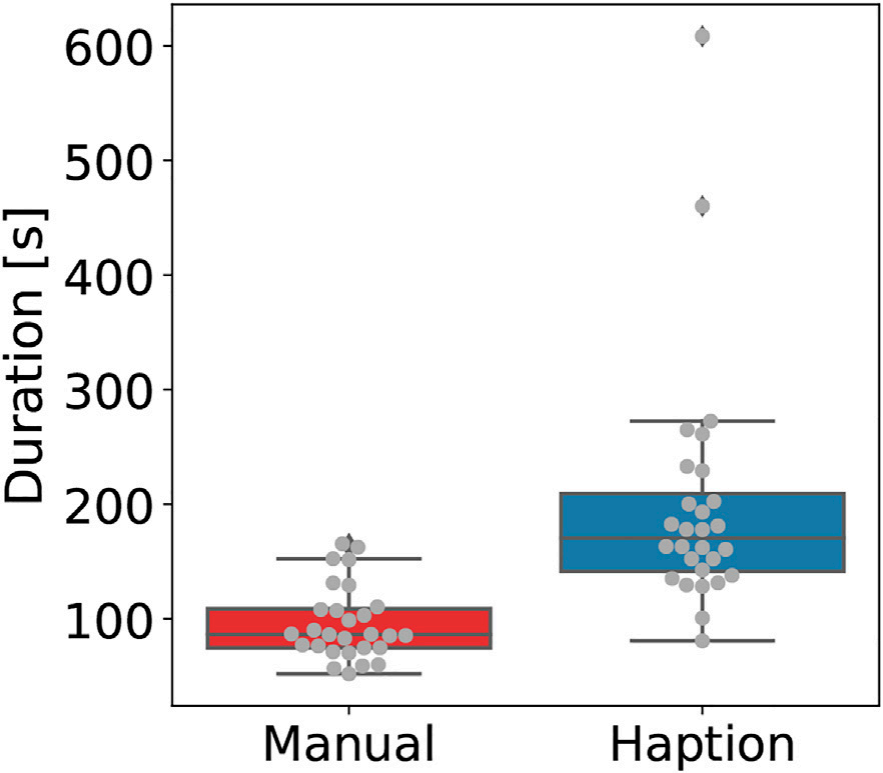
Boxplot with overlaid beeswarm plot comparing the duration per trial.

**FIGURE 6:**
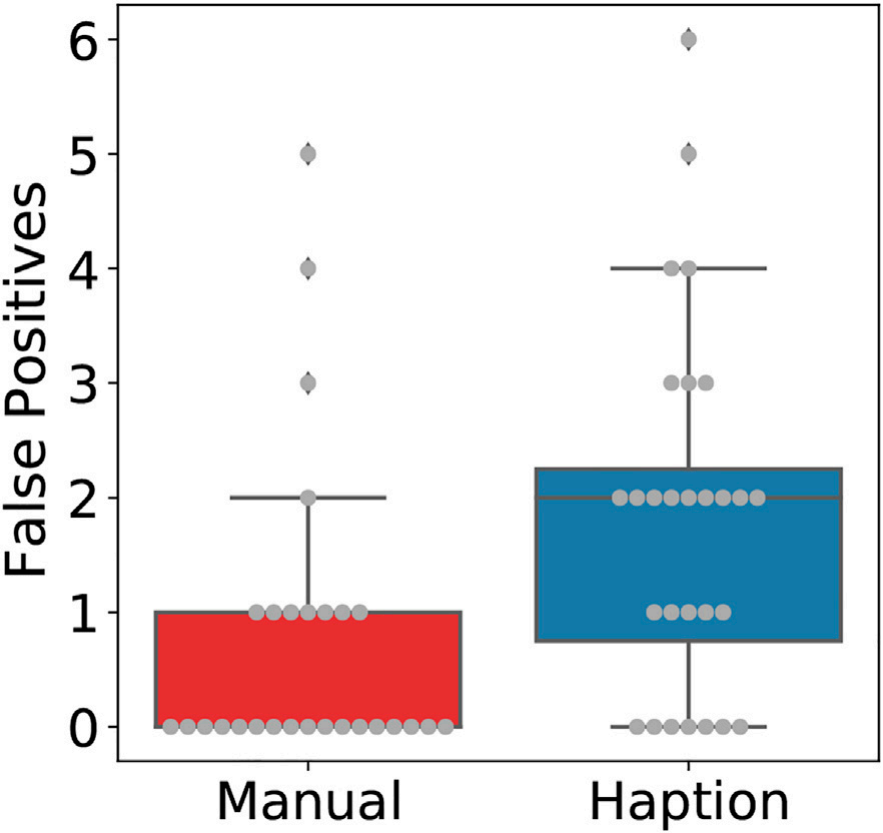
Boxplot with overlaid beeswarm plot comparing the number of false positives reported by test subjects per trial.

**FIGURE 7:**
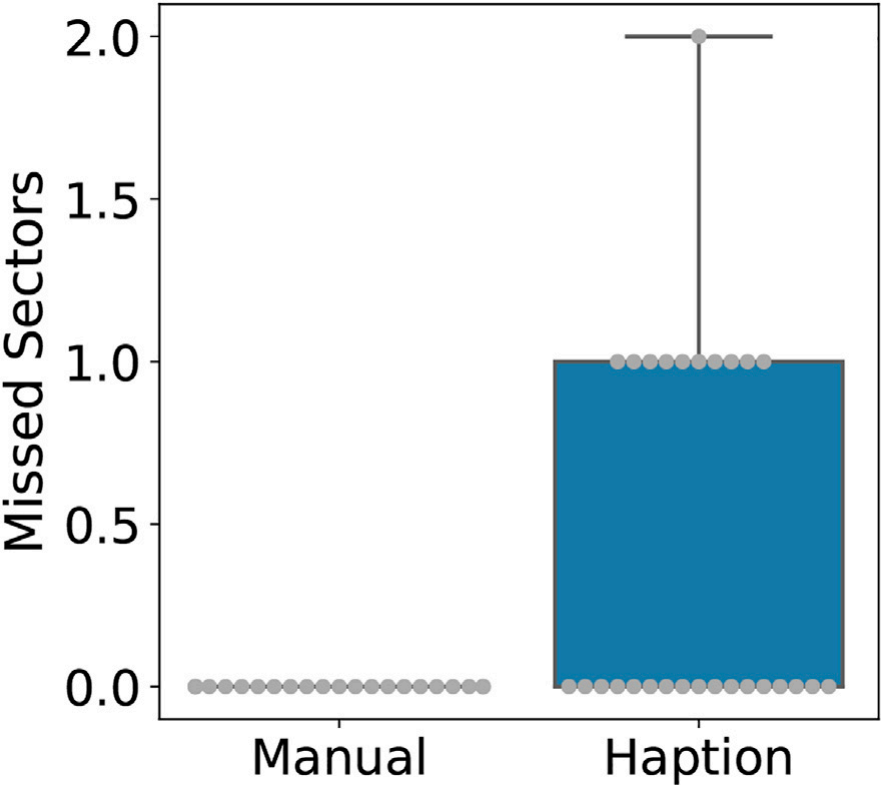
Boxplot with overlaid beeswarm plot comparing the number of contaminated tiles not reported per trial.

Experiments were conducted as described in Section 3.3. Seven test subjects participated in the experiment, six of them being trained remote handling operators and one novice user; this number was representative of the number of available remote handling operators on site. All the test subjects had experience with robots and teleoperation systems, such as the MASCOT system (Hamilton and Preece, 2001) used during operations in the Joint European Torus (JET) (Sanders, 2006) project. All the test subjects had training and experience using the haptic device and had a familiarisation period with the robot holding the sensor probe. All the test subjects managed to perform the radiation surveying task using both manipulation methods (i.e., they manipulated the sensor probe and found contaminants in the glovebox workspace). Only one iteration of the test had cross-contamination of the probe (i.e., the probe was in direct contact with the LS1 liquid), leading to resetting the test after appropriately cleaning the probe; this instance occurred during one tele-manipulation trial. The task metrics introduced in Section 3.3.4 were used to compare task performance between manipulation methods with two sample t-tests (Delacre et al., 2017). Table 1 shows the resulting task metrics averaged per manipulation method, and a more in-depth explanation of these results is provided next. Furthermore, these results are shown using boxplot figures (Figures 4–7) to express how sparsely distributed the answers were, compared to the average results.

**TABLE 1:** Averaged performance metrics per manipulation method.

Manipulation method	Completion time [s]	False positives	Number of missed sectors
Manual	96.11	0.75	0
Teleoperation	199.36	1.82	0.42

The number of trials where a collision occurred was significantly larger while using the local device of the tele-manipulation system, as seen in Figure 4. This difference is statistically significant with *p* = 1.83*d* − 05. This result is of significance considering that a single collision between the sensor and a contaminated area is enough to potentially damage the sensing probe and lose its measuring capabilities.

Figure 5 depicts the completion time in trials, where the duration for each trial was significantly longer and more inconsistent while using the tele-manipulation system. On average, the manual operation took 96.11 s, whereas the tele-manipulation system took 199.36 s to complete a trial. This difference is statistically significant, with *p* = 2.95*d* − 05 reported from a two-sample *t*-test with unequal variance, i.e., Welch’s *t*-test, (Delacre et al., 2017).

Regarding accuracy, both the number of tiles incorrectly detected as contaminated (i.e., false positives) and the missed sectors were significantly higher during tele-manipulation, with a significant difference on the latter as no missed sectors were reported during manual operation. Figure 6 shows the number of false-positive sectors reported per trial, with on average 1.82 sectors badly reported during the tele-manipulation against 0.75 during the manual operation; this is a statistically significant difference with *p* = 0.007. In contrast, Figure 7 shows the number of contaminated sectors that were not reported during trials, with 0 reported during the manual operation against 0.42 during the tele-manipulation on average; this difference is statistically significant with a *p* = 0.0004 reported from a two-sample *t*-test with unequal variance, i.e., Welch’s *t*-test.

**FIGURE 8:**
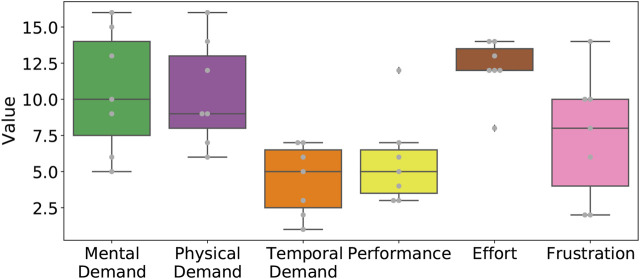
Boxplot with overlaid beeswarm plot of the reported NASA-TLX results.

The subjective experience of test subjects using the tele-manipulation device when compared to manual operation was recorded using the NASA-TLX test. Figure 8 summarizes these findings, with large variation between test subjects in the frustration, mental demand, and physical demand factors. Given that the NASA-TLX ranges from 1 to 20, the group average for these factors was medium but with large variability, which makes it difficult to draw conclusions from these factors. Temporal demand and performance show less variability and low average values, meaning most test subjects did not feel pressed or rushed to finish the task using the tele-manipulation system compared to manual operation. However, most test subjects reported high values in the effort factor (12 on average), which can be interpreted as the test subject having to put in significantly more effort to perform radiation surveying in the glovebox using the tele-manipulation system.

Although our study lacked sufficient test subjects to draw a statistically significant conclusion, task performance was not significantly worse off for the novice test subjects compared to expert operators. All trials took longer than the group average (above 260 s against 199.36 s), with half of the trials taking longer than 300 s; however, the occurrences of collisions, false positives, and missed sectors were similar to those of other test subjects. This was expected due to the difference in expertise using both teleoperation devices and performing radiation surveying.

There were no relevant performance improvements over trials per test subject (i.e., task performance did not improve steadily with repetition). Test subjects reported feeling more comfortable with the tele-manipulation interface, and there was no significant improvement, meaning the duration stayed above 100 s, and occasional collisions and missed detected sectors were observed.

**FIGURE 9:**
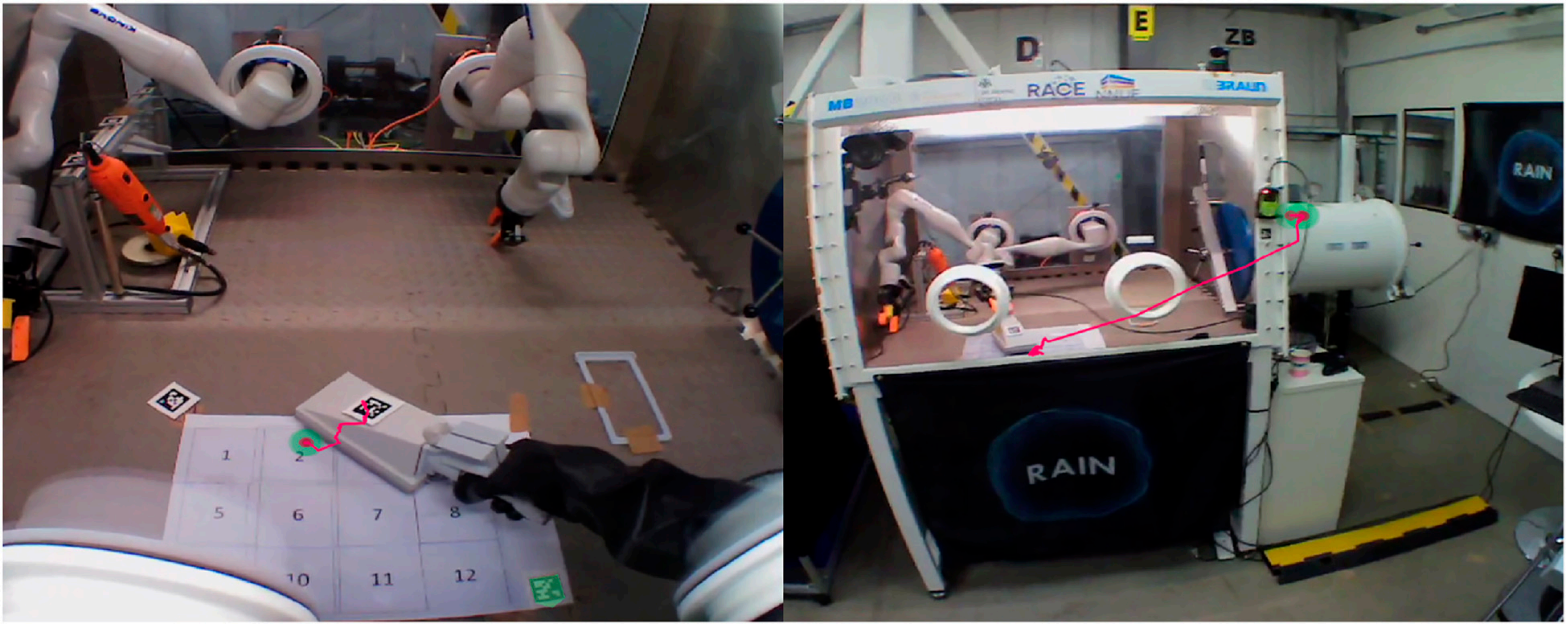
Example of fixations (green dot) and gaze patterns (purple lines) while performing radiation surveying using manual operation (left) and tele-manipulation (right).

Regarding gaze patterns and engagement with the task being performed, all test subjects focused on the scanning task using any of the manipulation methods (i.e., keeping fixation inside the workspace and around relevant equipment). A raster scanning pattern was seen in most test subjects, going side to side and changing rows one sector at a time. Fixations were either an onset of the sector to be scanned next or pursuing of the probe as it moves. In addition to workspace fixations, the radmeter was consulted to verify a contaminant being detected; however, most test subjects relied on the sound alarm produced by the radmeter alone and focused on the workspace, except for the novice user. Figure 9 shows the fixations and motion between fixations for a manual operation and a tele-manipulation trial, with the latter mimicking the manual operation until a contaminant was found. Other fixation points during trials were only seen during tele-manipulation, which were the Kinova second joint and the haptic device itself. Five out of seven test subjects changed gaze from the glovebox to the dials and controllers in the haptic device, either to use the redundancy buttons or to confirm grip and grasp inputs; three test subjects had to look at the haptic device in three trials, one on two trials and one during a single trial alone.

### 4.1 Remote handling operator interview

After completing the trials, the JET remote handling operators were asked the following questions, indicated with **Q**, and the answers to the interview questions, indicated with **A**, are summarized as follows:

**Q** The teleoperation system has haptic/force feedback for the teleoperator. Were you aware of the haptic feedback during the experiment? If you were aware, did you find it useful for completing the task? Why?
**A** All interview participants, except for one, indicated that they were aware of the force feedback in the bilateral teleoperation system. The majority of the answers clearly stated that they felt the weight of the sensor. All answers indicated that the force feedback is useful for tele-manipulation.
**Q** You have experienced two different manipulation methods for gloveboxes. Which method would you prefer if you were given the chance to select? Please do not take the radiation hazard into account when answering the question.
**A** All interview participants, except for one, preferred manual manipulation over tele-manipulation because it was considered more intuitive, quicker, and easier. One participant thought that manual manipulation is more exhausting, and the participant finds tele-manipulation easier to use.
**Q** Do you think that, from a user’s perspective, the teleoperation system can be improved? How?
**A** Three answers suggested using a teleoperation system with similar kinematics as the robots. Two answers highlighted the importance of the relative positioning and orientation of the local and remote robots. Two answers reported that the inverse kinematics algorithm used in the teleoperation systems should give more intuitive joint configuration. One answer commented on improving the clutching mechanism of the teleoperation system for a smoother transition after releasing the clutch. One answer suggested using auditory or visual feedback to the operator in case of reaching the limits of the physical workspace of the haptic interface. One answer suggested having higher fidelity in force feedback so that touching the remote environment with the sensor could be perceived by the operator. One answer suggested having a force scaling mechanism where heavier objects are felt lighter on the operator side.
**Q** What was the most challenging part of the manual manipulation?
**A** The answers were around the physical limitations imposed by the glovebox. This is either working in a limited working area, reduced reaching capability, or carrying a heavy object (i.e., sensing probe) for prolonged time in uncomfortable body positions.
**Q** What was the most challenging part of the tele-manipulation?
**A** The answers highlight that understanding the foreign kinematic structure of the local and remote robots was the most challenging part of the tele-manipulation. Related to this issue, one interview participant highlighted the importance of training for the setup. With training, the operators are expected to develop better intuition on how the remote robot moves. In this context, the elbow motion of the remote robot as a result of the redundancy was identified as an important problem of the manipulation system. The limited workspace of the local robot compared to the remote one was identified as another limitation of the system. Moving the remote arm without colliding with the remote environment was considered as easy as they expected. Finally, interviewees stressed out the relative orientation of the local and remote robots.

## 5 Discussion

As seen in Section 4, there is substantial evidence to support the claim that task performance during tele-manipulation was considerably lower than during manual operation. Although one might argue that it is not fair to compare the task performance of any teleoperation system or device with that of manual operation, it is necessary to adopt manual operation as the ground truth or basic performance that any future robotic system should achieve or outperform. The tele-manipulation system provided flexible and effective haptic-enabled control of the robot’s end-effector, but task performance was not ideal even when used by trained operators. A task-aware technical analysis is necessary to understand the reasons behind these results and ways to improve them. The next step is to analyze the results, provide our hypothesis for why task performance was so dissimilar, and propose solutions for future teleoperation systems.

The largest factors affecting the teleoperation performance are the same that define any task performed inside a glovebox: the interplay between the limitations of the manipulation method, environmental constraints, and tool characteristics. First, the glovebox is a restricted space, with visibility only available through shaded glasses placed on one side of the box. In addition, the sensor used during the trials is a realistic training probe that weighs 0.9 Kg, which was even reported as heavy during manual operation by some test subjects. Furthermore, the Kinova Gen 3 robot has a redundant joint (elbow joint) that allows reaching many end-effector configurations by rotating the redundant joint close to kinematic singularities, leading to potential collisions between the joint and the glovebox limits.

The large numbers of probe collisions were primarily due to the reduced depth perception inside the glovebox, which complicates the test subject’s task of estimating the distance between the probe and the floor; this estimation is crucial while moving the probe, as optimal radiation surveying requires constant distance and orientation relative to the floor. The weight of the sensor probe and the robot’s joint configuration necessary to use the sensor lead to further control difficulties. When starting control action from the haptic device using the enabling clutch and the dead man’s switch, a sudden and unplanned drop in the end-effector’s position occurred the closer the probe was to the floor, sometimes leading to collisions between the end effector and the glovebox’s floor. Although the weight of the sensor is well within the robot’s reported 2 kg full-range payload, it is theorised that the cantilever-like configuration required to operate the probe (see Figure 2A) is particularly challenging for the haptic device’s commercial weight compensating torque controller. All these translate to reduced dexterity, with big effort needed to stabilize and operate the haptic device, as reported in the NASA-TLX at the end of Section 4. It could be argued that redesigning the sensor probe or the gripping block to allow the sensor to be grasped and manipulated from a different angle (i.e., vertical orientation instead of a mostly horizontal) would help, but these ad-hoc solutions would restrict the robot’s workspace, its usability, and incur redesigning costs.

The situations described below can also explain the low accuracy experienced during tele-manipulation (i.e., the large number of tiles incorrectly detected as contaminated and missed sectors). Low dexterity makes it difficult to differentiate one sector from the next, as the probe requires slow and stable movements parallel to the glovebox’s floor.

It is worth noting that both collisions between the probe glovebox and contaminants not found are not acceptable during any radiation surveying task. Although short surveying times and no false positives are desired, long and overestimating survey are safer and more desired than surveys damaging the sensor probe or missing dangerous contaminants.

The experimental setup uses a simulated radiation source and a sensor probe that can detect it. All participants are informed before participating in the study about the simulated radiation source. The experimental setup is kept as realistic as possible; however, knowing the radiation source is simulated could encourage participants to be less cautious while operating the device. With a real radiation source, we expect operators to take extra precautions, potentially increasing their surveying times for both manipulation methods. Whether it is simulated or real radiation, the authors believe that the relative difference in performance between manual and tele-manipulation will remain.

The interviews with professional remote handling operators revealed interesting design pointers and helped researchers to have a better understanding of the expectations from industry professionals.

The answers of remote handling professionals reflect their experience with the JET remote handling system, and they are inherently biased to favor this system over other tele-manipulation systems. Their comments on force scaling and similar kinematics reflect this preference. However, this bias is not something researchers should ignore. On the contrary, the suggestion from the interviews about having a similar kinematic structure for both local and remote robots improves the operational safety and ease of use of the tele-manipulation system. Being able to control each link of the remote robot is crucial in this context.

The existence of force feedback is appreciated by the operators; however, as pointed out in an answer by an interviewee, the force rendering fidelity of the selected COTS teleoperation system did not allow operators to feel low amplitude contact forces; hence, force feedback was not utilized to secure a collision-free course for the radiation sensor. On the contrary, all operators relied on their vision to detect collisions. We deduce that the transparency of the tele-manipulation system is crucial for safe operations and, in this particular case, the agility of the operator. This hypothesis needs further evaluation.

Using a robot with different kinematic structures offers advantages such as reduced cost. However, the inverse kinematics algorithm becomes crucial for such tele-manipulation systems. In the presence of redundant manipulators, the inverse kinematics could significantly degrade the performance by causing redundant elbow motion. This phenomenon was detected by the participants and identified as a problem of the tele-manipulation system.

One participant identified an important problem regarding the haptic interfaces. As the operator is mentally engaged with the remote task during teleoperation, there is no way to distinguish the end of the physical workspace or a collision in the remote environment. In both situations, the haptic interface resists the motion of the operator. This situation creates confusion on the operators, and we argue that it increases the cognitive load on the operator.

Considering the problems shown during tele-manipulation using an advanced commercial solution, we present a list of key features and improvements necessary for control interfaces used in radiation surveying and other glovebox maintenance tasks:

• Cartesian motion and velocity compensation are used to move at a fixed distance from a surface while holding a fixed orientation.
• Collision avoidance between the robot joints, the sensor probe, and the glovebox itself.
• Introduce a constant-torque mode for the Haption device, which compensates for the payload weight and holds the robot’s end-effector position between activations.

Additional assisting technologies have been explored in the state-of-the-art to improve teleoperation systems’ performance overall, which include adding automated control sub-routines (Qi et al., 2021), including environment segmentation and object classification for grasping using cameras (Su et al., 2020b), intention reading of the operator (Oh et al., 2021), and other control-related technologies (Su et al., 2020a), that rely on sensor feedback and deep-learning or neural networks. However useful these technologies might be, the requirements for nuclear glovebox operations limit the adoption of these solutions. Adding sensors such as cameras inside nuclear gloveboxes is difficult and costly, with a reduced shelf life and limited performance observed in most cases. In addition, the use of black box solutions such as neural networks makes the creation of a safety case for a regulatory body such as the Office for Nuclear Regulation (ONR) in the United Kingdom a very complex problem. In future work, we will address this highly constrained and complex research challenge.

Finally, the authors would like to highlight that the experimental performance evaluation presented in this paper is very flexible in terms of assessing new manipulation interfaces or understanding the performance of different tasks. Assessing the performance of different manipulation interfaces, such as a new teleoperated robotic manipulation system, is trivial, as it only requires completing the experimental procedure. The evaluation of performance in new tasks requires further work on designing the experiment itself. For instance, understanding the pick-and-place performance in glovebox operations requires designing an experiment based on Fitts’ Law (MacKenzie, 1992) or peg-in-a-hole.

## 6 Conclusion

A methodology to compare tele-manipulation methods in a glovebox environment was presented, using a radiation surveying task and different performance metrics. Task performance was measured for the tele-manipulation system compared to manual operation while using ionising radiation simulator systems used in the industry. A Haption Virtuose™ 6D TAO Virtuose controlling a Kinova Gen3 arm was shown to be able to perform radiation surveying by teleoperation; however, measured task performance was significantly lower than that of manual operation. A list of reasons and solutions to these problems were presented. We managed to show the shortcoming of the current off-the-shelf commercial offering for glovebox operations, as the current iteration of this system is still not sufficient to replace manual glovebox operations.

When faced with constraints in technical challenges, it is easy to advocate for a complete redesign or change in the equipment used (i.e., robot, glovebox, and sensor). However, these experiments exemplify the current challenges faced by robotic glovebox operations and systems, as robust and flexible solutions are needed to fit both legacy equipment and build toward the robotic gloveboxes of the future. By implementing and measuring a relevant maintenance task involving tool handling and a defined workspace similar to what an operator would face in manual operation, relevant comparisons and limitations can be seen in teleoperation interfaces.

Future work includes testing more test subjects from both an expert and novice background and creating a human–robot interface (HRi) that implements some of the improvements described in Section 5, namely, limiting end-effector motion to a plane at a certain distance from the floor.

## Data Availability

The raw data supporting the conclusion of this article will be made available by the authors, without undue reservation.

## References

[B1] Akiyama M. (1996). Research and development on decommissioning of nuclear facilities in Japan. Nucl. Eng. Des 165 (3), 307–319.

[B2] Ben-Porat O. Shoham M. Meyer J. (2000). Control design and task performance in endoscopic teleoperation. Presence. (Camb). 9, 256–267. 10.1162/105474600566781

[B3] Cumbria B. (2019). Worker exposed to sellafield plutonium had skin removed. BBC News.

[B4] Delacre M. Lakens D. Leys C. (2017). Why Psychologists Should by Default Use Welch’s <i&gt;t&lt;/i&gt;-test Instead of Student’s <i&gt;t&lt;/i&gt;-test. rips. 30, 92. 10.5334/irsp.82

[B5] Garrec P. Friconneau J.-P. Louveau F. (2004). “Virtuose 6d: A new force-control master arm using innovative ball-screw actuators,” in Proceedings of osr2004–35th symposium on robotics, paris, France march.

[B6] Geiger L. Popp M. Farber B. Artigas J. Kremer P. (2010). “The influence of telemanipulation-systems on fine motor performance,” in Third international conference on advances in computer-human interactions, 44–49.

[B7] Ghosh A. Alonso Paredes Soto D. Veres S. Rossiter J. (2020). “Human robot interaction for future remote manipulations in industry 4.0,” in IFAC-PapersOnLine; international federation of automatic control (IFAC), 10223–10228.

[B8] Grasz E. Perez M. (1997). “Addressing nuclear and hostile environment challenges with intelligent automation,” in Tech. rep. (Lawrence Livermore National Lab. LLNL).

[B9] Hagemeyer D. McCormick Y. (2012). “Occupational radiation exposure report,” in Tech. rep. (Oak Ridge Inst. for Science and Education ORISE).

[B10] Hamilton D. Preece G. (2001). Development of the MASCOT telemanipulator control system. European Fusion Development Agreement. Project.

[B11] Harden T. Lloyd J. Turner C. (2009). “Robotics for nuclear material handling at LANL: Capabilities and needs,” in Tech. rep. (Los Alamos National Lab. LANL).

[B12] Hart S. G. Staveland L. E. (1988). “Development of nasa-tlx (task load index): Results of empirical and theoretical research,” in Advances in psychology (Elsevier), 52, 139–183.

[B13] Lastinger M. C. Verma S. Kapadia A. D. Walker I. D. (2019). “Tree: A variable topology, branching continuum robot,” in International conference on robotics and automation (ICRA), 5365–5371. 10.1109/ICRA.2019.8794463

[B14] Lawrence D. (1993). Stability and transparency in bilateral teleoperation. IEEE Trans. Rob. Autom. 9, 624–637. 10.1109/70.258054

[B15] Li R. Jensen J. Hill J. Bowersox J. C. (2000). Quantitative evaluation of surgical task performance by remote-access endoscopic telemanipulation. Surg. Endosc. 14, 431–435. 10.1007/s004640010075 10858465

[B16] Liu W. Su Y. Wu W. Xin C. Hou Z.-G. Bian G.-B. (2019). An operating smooth man–machine collaboration method for cataract capsulorhexis using virtual fixture. Future Gener. Comput. Syst. 98, 522–529. 10.1016/j.future.2019.01.032

[B17] Long P. Padir T. (2018). “Evaluating robot manipulability in constrained environments by velocity polytope reduction,” in Proceedings of the international conference on humanoid robots (humanoids), 1–9.

[B18] MacKenzie I. S. (1992). Fitts’ law as a research and design tool in human-computer interaction. Human–Computer. Interact. 7, 91–139. 10.1207/s15327051hci0701_3

[B19] Masubuchi S. Morimoto M. Morikawa S. Onodera M. Asakawa Y. Watanabe K. (2018). Autonomous robotic searching and assembly of two-dimensional crystals to build van der Waals superlattices. Nat. Commun. 9, 1413. 10.1038/s41467-018-03723-w 29650955 PMC5897399

[B20] Nuclear Decommissioning Authority (2021). “Nuclear decommissioning authority strategy,” in Tech. rep. (Nuclear Decommissioning Authority NDA).

[B21] Oh Y. Schäfer T. Rüther B. Toussaint M. Mainprice J. (2021). “A system for traded control teleoperation of manipulation tasks using intent prediction from hand gestures,” in 2021 30th IEEE int. Conf. Robot hum. Interact. Commun., 503–508. 10.1109/RO-MAN50785.2021.9515440

[B22] O’Neil B. Brabec C. Pryor M. (2012). “Hazardous workspace modeling for manipulators using spatial hazard functions,” in 2012 IEEE int. Symp. Safety, secur. Rescue robot., 1–6. 10.1109/SSRR.2012.6523880

[B23] O’Neil B. (2010). Graph-based world-model for robotic manipulation. University of Texas at Austin. Ph.D. thesis.

[B24] Onol A. Long P. Padir T. (2018). “Using contact to increase robot performance for glovebox d and d tasks,” in Proceedings of the waste management symposia.

[B25] Pan C. Liu X. Jiang W. (2017). “Design and synchronization control of heterogeneous robotic teleoperation system,” in 2017 Chinese autom. Congr, 406–410. 10.1109/CAC.2017.8242801

[B26] Pedrotti L. R. Armantrout G. A. Allen D. C. Sievers., R. H. Sr (1991). “Robot development for nuclear material processing,” in Tech. rep. (CA (United States): Lawrence Livermore National Lab.).

[B28] Perera D. Tucker J. W. Brahmbhatt S. Helal C. J. Chong A. Farrell W. (2018). A platform for automated nanomole-scale reaction screening and micromole-scale synthesis in flow. Science 359, 429–434. 10.1126/science.aap9112 29371464

[B29] Peterson K. D. (2000). “Robotic system for automated handling of ceramic pucks,” in Tech. rep. (Lawrence Livermore National Lab).

[B30] Qi W. Ovur S. E. Li Z. Marzullo A. Song R. (2021). Multi-sensor guided hand gesture recognition for a teleoperated robot using a recurrent neural network. IEEE Robot. Autom. Lett. 6, 6039–6045. 10.1109/LRA.2021.3089999

[B31] Richard P. Coiffet P. Kheddar A. England R. (1999). “Human performance evaluation of two handle haptic devices in a dextrous virtual telemanipulation task,” in Proceedings IEEE/RSJ international conference on intelligent robots and systems, 3, 1543–1548. Human and Environment Friendly Robots with High Intelligence and Emotional Quotients.

[B32] Roa M. Suárez R. (2015). Grasp quality measures: Review and performance. Auton. Robot. 10.1007/s10514-014-9402-3 PMC4457357 26074671

[B33] Rollow T. (2000). “Type a accident investigation of the March 16, 2000 plutonium-238 multiple intake event at the plutonium facility,” in Tech. rep. (Los Alamos National Laboratory).

[B27] Sands D. (2006). Cost effective robotics in the nuclear industry. Ind. Robot. Int. J 33 (3), 170–173(4). 10.1108/01439910610659079

[B34] Sanders S. (2006). Remote operations for fusion using teleoperation. Industrial Robot Int. J. 33, 174–177. 10.1108/01439910610659088

[B35] Sharp A. Hom M. W. Pryor M. (2017). “Operator training for preferred manipulator trajectories in a glovebox,” in 2017 IEEE work. Adv. Robot. Its soc. Impacts., 1–6. 10.1109/ARSO.2017.8025193

[B36] Siciliano B. Sciavicco L. Villani L. Oriolo G. (2008). Robotics: Modelling, planning and control. Springer.

[B37] STS Ltd (2022). “RadEye DP6 simulator,” in Tech. rep. (Safe Training Systems STS).

[B38] Su H. Hu Y. Karimi H. R. Knoll A. Ferrigno G. De Momi E. (2020a). Improved recurrent neural network-based manipulator control with remote center of motion constraints: Experimental results. Neural Netw. 131, 291–299. 10.1016/j.neunet.2020.07.033 32841835

[B39] Su H. Qi W. Yang C. Sandoval J. Ferrigno G. Momi E. D. (2020b). Deep neural network approach in robot tool dynamics identification for bilateral teleoperation. IEEE Robot. Autom. Lett. 5, 2943–2949. 10.1109/LRA.2020.2974445

[B40] Tokatli O. Das P. Nath R. Pangione L. Altobelli A. Burroughes G. (2021). Robot-assisted glovebox teleoperation for nuclear industry. Robotics 10, 85. 10.3390/robotics10030085

[B41] Tonsen M. Baumann C. K. Dierkes K. (2020). A high-level description and performance evaluation of pupil invisible. arXiv preprint arXiv:2009.00508.

[B42] Turner C. Pehl J. Cox D. Tesar D. (2001). “Design of small automation work cell system demonstrations,” in American nuclear society proceedings of the 9^th^ topical meeting on robotics and remote systems.

[B43] Wagner C. R. Howe R. D. (2007). Force feedback benefit depends on experience in multiple degree of freedom robotic surgery task. IEEE Trans. Robot. 23, 1235–1240. 10.1109/tro.2007.904891

[B44] Wang Z. Reed I. Fey A. M. (2018). “Toward intuitive teleoperation in surgery: Human-centric evaluation of teleoperation algorithms for robotic needle steering,” in 2018 IEEE int. Conf. Robot. Autom., 5799–5806. 10.1109/ICRA.2018.8460729

[B45] Xu X. Cizmeci B. Schuwerk C. Steinbach E. (2016). Model-mediated teleoperation: Toward stable and transparent teleoperation systems. IEEE Access 4, 425–449. 10.1109/ACCESS.2016.2517926

[B46] Yip M. C. Tavakoli M. Howe R. D. (2011). Performance analysis of a haptic telemanipulation task under time delay. Adv. Robot. 25, 651–673. 10.1163/016918611x558216

